# A Comprehensive Review on Infrared Heating Applications in Food Processing

**DOI:** 10.3390/molecules24224125

**Published:** 2019-11-15

**Authors:** Salam A. Aboud, Ammar B. Altemimi, Asaad R. S. Al-HiIphy, Lee Yi-Chen, Francesco Cacciola

**Affiliations:** 1 Department of Food Science, College of Agriculture, University of Basrah, Basrah 61004, Iraq; salamalmiyahi@gmail.com (S.A.A.); aalhilphy@yahoo.co.uk (A.R.S.A.-H.); 2 Department of Plant Soil and Agricultural Systems, Agriculture College, Southern Illinois University, Carbondale, IL 62901, USA; yclee010689@gmail.com; 3 Department of Biomedical and Dental Sciences and Morphofunctional Imaging, University of Messina, 98125 Messina, Italy; cacciolaf@unime.it

**Keywords:** infrared, heating, microwave, cooking, conductive heating

## Abstract

Infrared (IR) technology is highly energy-efficient, less water-consuming, and environmentally friendly compared to conventional heating. Further, it is also characterized by homogeneity of heating, high heat transfer rate, low heating time, low energy consumption, improved product quality, and food safety. Infrared technology is used in many food manufacturing processes, such as drying, boiling, heating, peeling, polyphenol recovery, freeze-drying, antioxidant recovery, microbiological inhibition, sterilization grains, bread, roasting of food, manufacture of juices, and cooking food. The energy throughput is increased using a combination of microwave heating and IR heating. This combination heats food quickly and eliminates the problem of poor quality. This review provides a theoretical basis for the infrared treatment of food and the interaction of infrared technology with food ingredients. The effect of IR on physico-chemical properties, sensory properties, and nutritional values, as well as the interaction of food components under IR radiation can be discussed as a future food processing option.

## 1. Introduction

Heating is one of the important thermal processes in food processing and depends on the transfer of heat by conduction, convection, and radiation [1]. The goal of heating food is to increase its shelf life [2]. Traditional heating is either by burning fuel or by using electrical source. Heat is transferred from the outside to the surface of the food through convection and conduction and the temperature of the surface of the food increases even more until the heat reaches through the conduction into the food. When the temperature increases more, it causes physical and chemical changes of the heated substance [3].

Infrared is part of the electromagnetic spectrum that is located between the visible region and microwaves, and its wavelength ranges from 0.5 to 100 µm. Infrared rays are divided into three types: Near-IR (NIR) whose wavelength ranges from 0.75 to 1.4 µm at temperatures below 400 °C, and mid-IR (MIR) whose wavelength is between 1.4 and 3 µm at temperatures between 400 and 1000 °C and far-IR radiation (FIR) wavelength of 3–1000 µm at temperatures above 1000 °C [4,5]. Infrared penetration causes vibrating movement of water molecules at a frequency of 60,000–150,000 MHz and thereby causes heating. The dissipation of radiant energy in the form of heat produces a warming of the surface of the food and the penetration depth depends on the thickness of the product, the water activity, the product components and the wavelength of the infrared radiation [4]. Pan and Atungulu [6] described the benefits of infrared technology. Infrared technology is characterized by high energy efficiency, less water consumption, and environmental friendliness. In addition, it is also characterized by the homogeneity of heating with high heat transfer rate, low heating time, low energy consumption, and improving product quality and food safety. It also features low energy costs and the size of infrared equipment is small as well as controlling factors with high accuracy [7]. Moreover, it possesses unique radiation properties. The thermal efficiency of IR is high, and it is considered a valuable energy source. Gary Zeman who is a certified health physicist and the spokesman for the Health Physics Society pointed out that based on a study conducted by the National Research Council, there was no link between infrared cooking and cancer in an interview with The Jamaican Gleaner in 2008. The study also indicated that infrared radiation did not have enough energy to halve or damage DNA [8]. The energy is directly concentrated on the material to be heated and does not produce volatile organic compounds, carbon monoxide, or nitrogen oxides. It does not need heat recycling and does not need an isolated system. Nindo and Mwithiga [9] stated that infrared radiation does not pollute the environment compared to fossil fuels. In addition, it preserves vitamins, has very little flavor loss, and no heating of the surrounding air [5,10,11]. The Food and Drug Administration (FDA) has indicated that infrared radiation can be used in food processing, which is not ionizing. However, Mortensen [8] stated that care must be taken when using infrared radiation because it produces high heat, causes burns, low penetration depth of foods, and long-term exposure to infrared radiation causes tissue rupture [2]. 

Numerous studies have indicated that infrared possessed efficient effects in many manufacturing processes. Anagnostopoulou et al. [12] found that the total phenols found in infrared dried orange peel were higher compared to the dried orange peel using hot air. Many researchers have reported that infrared radiation improves the quality of food [13,14] and inhibits enzymes [15]. Infrared was used in the bakery because of its high efficiency in heat transfer processes [16]. In order to further increase its efficiency and improve energy use, microwave and infrared radiation were used together in roasting hazelnuts [17]. In addition, infrared radiation can be used to heat liquid food such as lemon juice [18].

Infrared can be used in numerous ways, such as stabilization of immature rice grain [19], pre-heating of drying [20,21,22], peeling [23], polyphenol recovery [24], freeze-drying [25], refractance window drying [26], antioxidant development [27], microbiological inhibition [28], sterilization infrared grains [29], baking bread [30], roasting food [31], manufacture of juices [18,32], and cooked food [33,34]. The aim of this comprehensive review is to investigate and discuss the effect of IR on sensory, nutritional, microstructural quality of foods and physico-chemical changes. This review emphasizes on opportunities and limitations in the processing of infrared foods, which can be possibly explored.

## 2. Infrared Radiation

**Figure 1:**
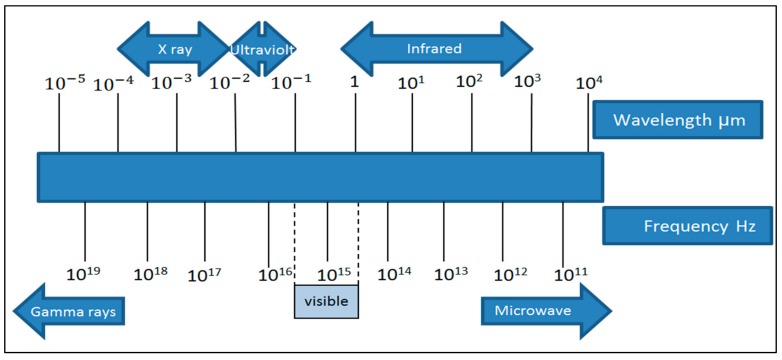
Electromagnetic radiation spectrum.

**Figure 2:**
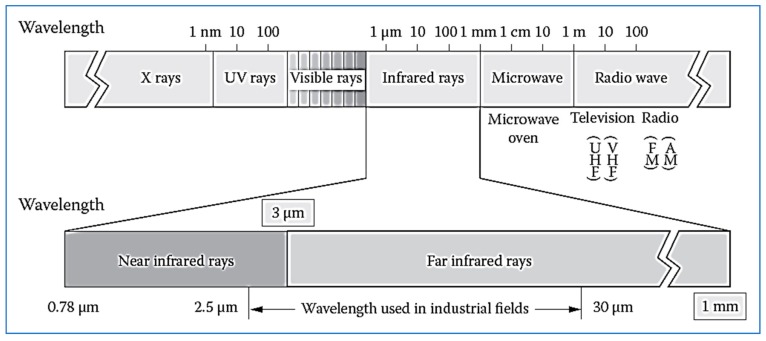
Spectrum of electromagnetic waves and infrared ranges.

Infrared radiation is part of the electromagnetic spectrum that is located between the visible region and microwaves. Its wavelength ranges from 0.5 to 100 µm. In 1800, Herschel wrote the first scientific report on infrared heating. In his report, it was shown that when the lenses were exposed to the sun, the paper beneath was burned. In the study, a prism was used to separate the light into different colors and a thermometer was subsequently placed in the different colors of the solar energy spectrum. Researchers noticed that there was severe heating in the area beyond the red color, which began at the end of the visible spectrum. The invisible part of the spectrum is called infrared and it is in the wavelength range of 0.76–350 µm [3]. The heat from the sun’s rays is infrared and is part of the electromagnetic radiation [6]. Sixty-six percent of solar radiation is infrared. Figure 1 and Figure 2 illustrate the electromagnetic spectrum of different types of radiation, including infrared.

The wavelength *λ* is the inverted frequency *ν* and can be calculated according to the following equation:

(1) $$ʎ=\frac{c}{v}\;or\;\frac{1}{ʎ}=\frac{v}{c}=\frac{v}{3\times 10^{10}}$$

where *c* is the speed of electromagnetic radiation (cm/s).

The wave number can be calculated from the following equation and its units (1/cm)

(2) $$\bar{v}=\frac{1}{ʎ}$$

*ΔE* can be calculated from the following relationship:

(3) $$\Delta E=h\bar{v}c$$

The conversion between wavelength and wavenumber is:

(4) $$ʎ\;\left(\mu m\right)=\frac{10000}{\bar{v}\;\left(cm^{-1}\right)}$$

The maximum obtained power at a given wavelength µm and *T* divided by the unit area (cm^2^) of the black body is calculated as follows:

(5) $$I_{max}=1.290\times 10^{-15}T^{5}\;\left(Wcm^{-2}\mu m^{-1}\right)$$

The Stefan–Boltzmann function shows the relationship between the intensity of the emitted radiation (*I*) and the absolute temperature (*T*) of the source as in the following equation:

(6) $$I=5.679\times 10^{-12}T^{4}\;\left(Wcm^{-2}\right)$$

Infrared radiation is divided into three types [3]:

1. Near-IR (NIR) with wavelength ranging from 0.75 to 1.4 µm.
2. Mid-IR (MIR) with a wavelength between 1.4 and 3 µm.
3. Far-IR radiation (FIR) with wavelength between 3 and 1000 µm.

Therefore, infrared radiation is defined as part of an electromagnetic spectrum whose wavelength ranges from 0.75 to 1000 µm.

Infrared heating depends on the spectrum because the energy emitted from the emitter consists of different wavelengths and part of the radiation depends on the source temperature and the lamp emission. The phenomenon of radiation becomes more complex because the amount of radiation that falls on any surface depends not only on the spectrum, also on the direction. Electromagnetic radiation is weakened as a result of absorption by the medium as well as scattering. The process of converting radiation to other forms of energy is a phenomenon of absorption, while in the case of scattering, the radiated energy is directed to another destination from the original direction of propagation as a result of the combined effect of reflection, refraction, and deviation, and all these factors cause weak electromagnetic radiation [13,34].

**Figure 3:**
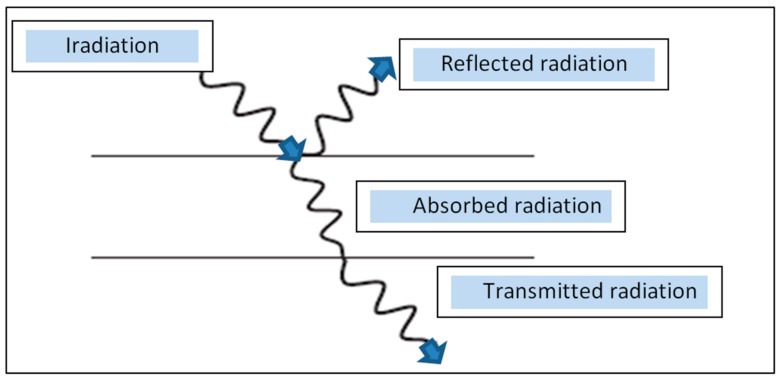
Extinction of radiation [6].

The radiation falling on a particular object is converted to heat and the energy can be absorbed and reflected, in addition, the radiation can be absorbed and transmitted as shown in Figure 3. This figure shows that there are three basic radiation properties, they are reflectivity (*ρ*), which is the ratio of the reflected part of the radiation coming to radiation next macro. Absorptivity (*α*), which is the ratio of the absorbed portion of incoming radiation to total incoming radiation. Emissivity transmissivity (*τ*), which is the ratio of the emitted part of the incoming radiation to the total incoming radiation, and the energy balance is shown in the following equation:

(7) α+ρ+τ=1

### 2.1. Advantages and Disadvantages of Infrared Rays

Pan and Atungulu [6] stated that infrared technology is highly energy-efficient, less water-consuming and environmentally friendly and is also characterized by the homogeneity of heating, high heat transfer rate, low heating time, low energy consumption, improved product quality, and food safety. Other features include low energy costs, air is transparent to infrared, and the size of infrared equipment is small as well as controlling factors in it with high accuracy. It also has unique radiative properties and high thermal efficiency, and is considered an alternative source of energy and heating [2]. Gary Zeman who is a member of the National Research Council of America and the Association of Health Physics, declared that there was no relationship between cooking with infrared radiation and cancer. The study also pointed out that infrared radiation did not have enough energy to halve or damage DNA. The energy is directly concentrated on the material to be heated and does not produce volatile organic compounds, carbon monoxide or nitrogen oxides. It does not need heat recycling and does not need an isolated system [8]. 

Pan and Atungulu [6] noted that infrared radiation improves the quality of the treated product and improves the safety of treated food. It reduces the consumption of chemicals and water and also increases manufacturing efficiency. Nindo and Mwithiga [9] stated that infrared radiation did not cause environmental pollution compared to fossil fuels. In addition, this processing preserves vitamins and has very little flavor loss, and the temperature of the surrounding air is not affected [5,10,11]. Moreover, infrared heaters are environmentally friendly and produce neither NOx nor CO. The Food and Drug Administration (FDA) has indicated that infrared radiation can be used in food processing, which is non-ionizing. Among of all advantages of infrared technology, the scientist illustrated some disadvantages such as people have to be cautious when using infrared radiation because it produces high heat and exposure to cause burns, the depth of penetration of food is small and long-term exposure to infrared radiation causes tissue rupture and is not sensitive to the reflection properties of coatings [2,8].

### 2.2. Infrared Sources

Infrared energy emits from the sun as well as from any fire, no matter how small. Any substance above absolute zero temperature emits infrared radiation. The quality and intensity of the radiation are related to the temperature of the radioactive material under the Stefan–Boltzmann law [6]. Rosenthal [3] illustrated that most sources of light and heat, including the sun, give some energy in the infrared range. When heated to a temperature below 870 K, the black body emits infrared radiation. In addition, the stone furnace and electric heater give bright radiation, so the temperature of radioactive objects increases and the radiated energy turns into short wavelengths.

Two traditional sources of infrared production used for heating are electric heaters and gas-fired heaters. These methods produce infrared radiation in a range of 343–1100 °C for gas and 1100–2200 °C for electric heaters [35]. In order to prevent products from burning, the typical infrared temperature produced is between 650 and 1200 °C. The cost of gas heaters is very high but their operating cost is relatively low compared to the systems producing electrically infrared radiation (electric heaters). Infrared electric heaters are more widespread than gaseous because of their ease of control, fast heating rate, and clean energy. Infrared emitters are more flexible in producing the required wavelength for a particular application. Infrared radiation can also emit wavelengths ranging from short to long wavelengths depending on the voltage applied to the emitters. The efficiency of the operation of electric infrared heaters ranges from 40% to 70%, while the infrared gas heaters produce 30%–50% [35] and emit medium to long wavelengths. The suitable area for industrial heating ranged from 1.17 to 5.4 µm, which corresponds to temperatures of 260–2200 °C [33]. The best efficiency of heat transfer by radiation occurs when the heater turns red.

**Figure 4:**
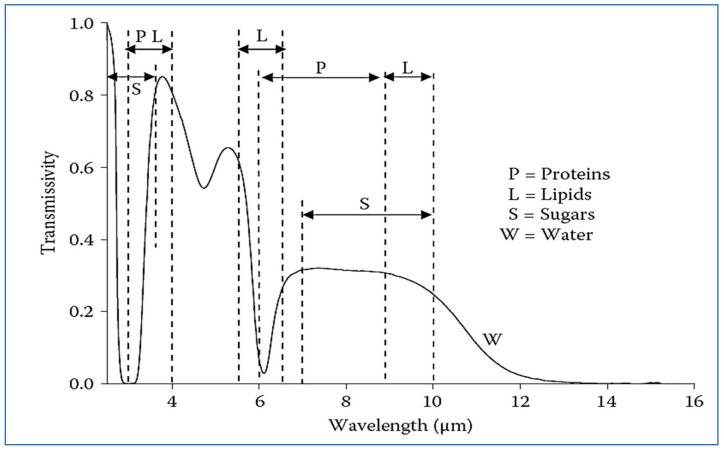
Principal absorption bands of the main food components compared with water [13].

Electrical infrared emitters (Figure 4) consist of a metal filament placed inside a sealed container and filled with inert gas or empty. Infrared radiation is produced using an electric heater by passing an electric current through a high-resistance wire such as nichrome wire, iron-chromium wire, and tungsten filament. When the metal wire is heated and reaches the glow temperature and its temperature rises to 2200 K, this will lead to the emission of infrared radiation type NIR wavelength ranging between 0.7 and 1.4 µm. Incandescent lamps that emit infrared radiation are classified as short-wave emitters, while quartz tubes and resistance elements are classified as medium- and long-wave emitters, respectively.

## 3. Infrared Food Heating Mechanism

Energy conservation is one of the factors that determine the usefulness and success of the operation of any food industry unit. Heat is transmitted by conduction, convection, and radiation. The goal of heating food is to increase the shelf life and improve the taste of foods [2]. Temperature is a measure of thermal motion at the molecular level. When the temperature of the material increases, the molecular motion gains more energy, and when it increases more, it causes physical and chemical changes in the heated material. In conventional heating, which comes from the combustion of fuel or electric heaters, heat is transferred to the material from the outside by convection by hot air or by thermal conduction. The process of transferring energy from source to food depends on the type of cooking. For example, in the case of the baking process, the energy is transmitted through convection, while frying and boiling are through conduction. Energy will be very close to the surface of the food and then heat food gradually from the hot surface towards the inside. Heat is transferred to the food through conduction only and this requires continuous processing of heat. The high temperature and time required for food depend on the thermal and engineering properties of the food [3].

When heating is done by radiation, the heat is transferred by convection and conduction. The broiling process takes place due to thermal radiation. Electromagnetic radiation causes thermal movements of the molecules, but conversion efficiency is highly dependent on the frequency (energy) of the radiation. Radiation-transmitted energy at shorter wavelengths than infrared causes electron-chemical changes in radiation-absorbing molecules, such as chemical bonding, electronic excitation, and dissipation of absorbed energy in the form of less heat. The efficiency of converting absorbed energy into heat is great at high wavelengths in infrared radiation, so the electromagnetic radiation produced by infrared radiation deepens the food by a few millimeters. Infrared radiation is absorbed by organic matter at separate frequencies that correspond to the transport of internal molecules between energy levels. This transition within the range of infrared energy is expressed regarding the rotational movement and the vibrational (stretching) movement of internal atomic bonds. The rotational frequencies range from 1011 to 1013 Hz with a wavelength of 30 µm^−1^ mm. The energy transfer during the separation of liquids is very small, and therefore, infrared absorption is continuous. Infrared absorption bands associated with food heating are shown in Table 1.

Table 1 shows that there is a strong absorption due to longitudinal vibrations. The absorption of the material to the radiation does not make it saturated with infrared radiation because the molecules excited by the vibratory movement continuously lose energy in random directions as a result of collisions between the molecules, which transfer energy to the surrounding environment in the form of heat. Wavelengths ranging within 1.4–5 µm are considered more effective in cooking food because of their ability to penetrate the steam layer surrounding the food as well as within the food a few millimeters deep. Most infrared radiation is absorbed by a thin layer of organic matter and water, so heating is superficial. The process of infrared heating is faster because the energy is transferred from the heating source to the food simultaneously. Therefore, there is no need for another method to transfer energy, for example, the use of hot air. The heat from infrared heating is produced on the surface of the infrared treated material, so the inside of the material is heated by the connection between the food molecules, thus the temperature is graded from the surface to the center. The air in contact with the surface of the food is heated indirectly, but it is not as hot as it occurs in heating by convection and conduction. The infrared absorption ranges by food components are shown in Figure 4, which shows that the food components interfere with each other in the absorption of different infrared spectra. Water mainly affects the absorption of incident radiation at all wavelengths, while the absorption of proteins by infrared radiation is at wavelengths 3–4 and 6–9 µm. Fat absorption is at wavelengths 3–4, 6 and 9–10 µm, and sugars are 3 and 7–10 µm. The water absorption beams are 3, 4.7, 6, and 15.3 µm [13]. In addition, when the thickness of the food increases, the absorption increases. 

### 3.1. Infrared Heating in Food Drying

Infrared wavelengths range from 2.5 to 200 µm and are often used in food drying processes. Water is strongly absorbed by infrared energy at wavelengths 3, 6, 12, and 15 µm [36,37]. Ceramic heaters are often used for drying processes because their emission is up to 3 µm. The reason why water absorbs infrared radiation strongly is the presence of O-H bonds in water, thus it begins to circulate at the same frequency of radiation. The process of converting infrared radiation into circulation energy causes water to evaporate. When infrared radiation hits the surface, part of it is absorbed, reflected, and transmitted. If the permeability is too small, the material reflects or absorbs infrared radiation depending on the nature of the radiation and the properties of the surface of the material and this is called emissivity (ε).

**Figure 5:**
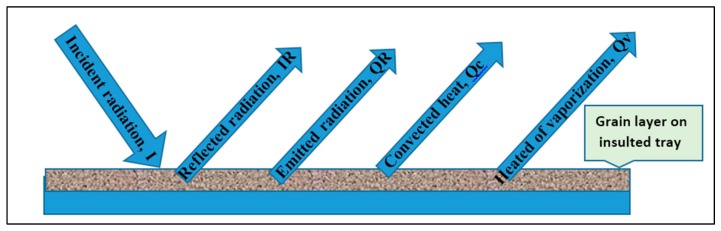
Energy balance on a thin layer of rough rice exposed to IR radiation.

The energy that dehydrates food is radiant energy. The infrared source used in food drying is infrared lamps and ceramic heaters by electricity or gas. Infrared rays do not need a medium to transmit radiation energy from the source to the surface of the food. This is an excellent feature, as the food is considered to absorb the infrared radiation and dry itself directly. Therefore, in order to improve the drying efficiency, the absorption and dispersion of the incident radiation should be lower and food should contain water. The infrared source must be in a closed room and its surface should be highly reflective for the purpose of maximizing the multiple reflections to increase energy efficiency [9]. Infrared absorption in food is differentiated regarding protein, fat, carbohydrates, and water. The direction of incident radiation, the properties of the food surface, and the spectral structure also determine the absorption of infrared radiation. One of the determinants of the use of infrared radiation in food is the heterogeneity of its shape and size, so the intensity of radiation falling on the material is different from one place to another. Figure 5 shows the transformation of fell IR on rice grains into different components [38]. The walls and bottom of the plate should be coated with aluminum foil in order to reduce heat loss and to reflect the falling rays on them and be radioactive walls. The increase of reflected and emitted radiation, heat transfer by convection and heat of evaporation is different depending on the surface characteristics and the water condition in the rice [36,38].

The natural vibration of the water molecule is in two cases, namely, symmetrical stretching vibration and symmetrical deformation vibration. Infrared energy relative to those frequencies is efficiently absorbed by the body. Therefore, the food absorbs infrared radiation efficiently at wavelengths greater than 2.5 µm through the change in the vibration state of the mechanism of vibration, which causes its temperature rise (heating) [39]. Richardson [40] noted that there are two basic vibrations: Stretching and bending, and expansion means increasing or decreasing the distance between the atoms and bending means the movement of atoms. When infrared radiation strikes molecules, energy will be absorbed and the vibration changes.

Laohavanich and Wongpichet [41] stated that the drying curve of rice at a wavelength of 2.7 µm is a function of drying time at initial moisture contents of 0.22, 0.27, 0.32, and 0.37 based on solid db weight, while moisture content 0.37 is a function of drying time at wavelengths of 2.47, 2.58, and 2.7 mµ. The moisture content decreases exponentially with the drying time and also shows that there is a significant effect of wavelengths on the drying rate of rice. The drying rate increases with increasing infrared wavelength. Drying time decreases with increasing wavelength.

Combining infrared and hot air is more efficient than if it was used individually as a result of their collaborative effect. Afzal et al. [11] found that when infrared and hot air were combined to dry barley, the energy consumption was reduced with good quality of barley. The use of infrared radiation with hot air reduces the total energy requirement by 245% compared to hot air alone.

### 3.2. Influence of Infrared on Antioxidants in Foods

#### 3.2.1. Total Phenolic Content

**Figure 6:**
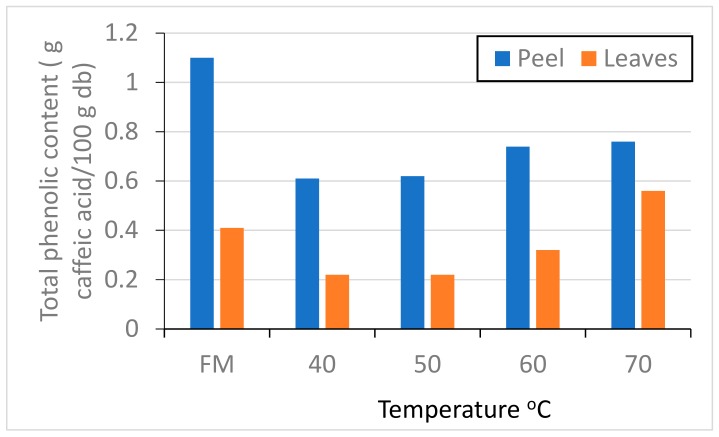
Effect of infrared temperature on total phenols of orange peel and leaves.

Phenolic compounds are antioxidants extracted from plants [42]. They have the ability to donate hydrogen or electrons as well as make the free radicals more stable [43,44]. The external peels of plants contain a large amount of phenolic compounds for the purpose of protecting their internal parts. Figure 6 shows the effect of infrared radiation at different temperatures on the total phenol content of orange peel and orange leaves. Fresh orange peel had a higher phenolic content compared to leaves. Infrared radiation has a significant effect on the peel and leaf content of total phenols. Plant cell components in the desiccant materials adhere to each other, and thus the possibility of solvent to extract the bioactive compounds will be more difficult [45]. When infrared treatment at high temperatures (60 and 70 °C) at a short period of time, the peel and leaf content of total phenols were higher because phenolic compounds resist thermal breakdown, as shown in Figure 6. The long drying time at low temperatures (40 and 50 °C) leads to the destruction of some phenols [46]. Anagnostopoulou et al. (2006) found that total phenols in infrared-dried orange peels were higher than in hot-air-dried [12]. Infrared rays can reactivate the low molecular weight antioxidants because heating the materials will be without damaging the underlying molecules of the heated surface and also contribute to heat transfer to the center of the heated material [47]. The effectiveness of the phenolic content increased after exposure of rice husks to FIR [48,49]. Lee et al. [50] found that exposure of rice husks to infrared radiation for two hours increased the content of phenolic compounds. When the rice husks are exposed to infrared radiation, the covalently linked phenol compounds that have antioxidant activity are released and activated.

Lee, et al., [2] showed that the total phenol content of an aqueous extract of peanut shells increased significantly when infrared exposure time and thermal treatment time were increased (Table 2). Total phenols increase from 72.9 µM for standard treatment (0) to 141.6 µM for infrared and 90.3 µM for conventional heating at 150 °C for 60 min. Infrared FIR is, therefore, more efficient in increasing phenol content in peanut shells compared with conventional heat treatment. Infrared radiation is biologically active [51], and heat is transferred evenly to the center of matter without breaking down surface-forming molecules [47]. Infrared may be able to access covalent bonds and release antioxidants [47,48]. On the other hand, simple heat treatment has increased the phenol content in the defat sesame, as well as citrus peel [52]. This shows that the association of phenolic compounds in plants is different depending on the type of plant. Effective manufacturing steps to release antioxidants from different plants may not be the same.

#### 3.2.2. Free Radical Scavenging 

When exposing the aqueous extract of peanut husks to FIR for 60 min, the percentage of free radical capture increased from 2.34% to 48.33%. In contrast, simple heat treatment increased to 23.69%. The increase depends on the time of exposure to both infrared and conventional heating [48,51]. 

The effectiveness of antioxidants was higher using infrared radiation with the initial treatment (pre-treatment with 5% potassium carbonate and 0.5% olive oil for 2 min at 20 °C) compared with standard treatment (infrared only) at 62 and 88 W (Table 3). The antioxidant efficacy of standard treatment at 125 W was higher than that of infrared treatment with the initial treatment. Therefore, in order to increase the effectiveness of antioxidants, the infrared capacity during drying should be reduced [53].

#### 3.2.3. Peroxide Value

**Figure 7:**
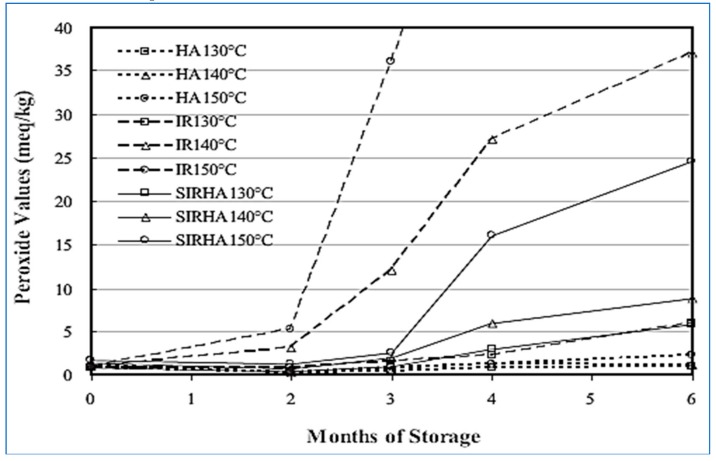
Changes in peroxide value of roasted almonds with IR and IR with hot air during storage at 37 °C [54].

The value of peroxide increases rapidly when only infrared and infrared with hot air are treated together as a result of higher temperatures. The value of peroxide after three months was 1.59, 12.10, and 36.07 meq/kg at temperatures of 130, 140, and 150 °C, respectively (Figure 7). Infrared roasting at 150 °C gives a significant increase in peroxide value and higher oxidation rates than other treatments. The reason for this was that the infrared rays penetrate the almonds quickly and cause the fat to move to the surface exposed to high temperature, thus causing rapid oxidation. The best conditions for roasting almonds and ensuring that the peroxide number of almonds within the permissible limits of 5 meq/kg are the use of infrared and hot air together and hot air only at temperatures of 130–150 °C, and the use of infrared radiation at 130 °C prolong the duration storage from four to five months at 37 °C, while hot-air roasting prolongs the storage period even longer [54]. Infrared roasting of cashew nuts improves the oxidative stability of its oil [55]. This may be the result of the formation of the products of Millard reactions that have antioxidant effects. 

#### 3.2.4. Tocopherol (Vitamin E)

Tuncel et al., [56] showed that the flaxseed content of γ and δ-tocopherol (flax does not contain α and β-tocopherol) for fresh and roasted infrared seeds was 146.57–193.14 and 2.91–3.23 mg/100 g, respectively. The effect of infrared on δ tocopherol was not significant, while the amount of γ tocopherol was high compared to fresh. The reason for obtaining the highest contents of γ tocopherol by infrared heating was the rupture of the cell walls by heat treatment, which led to increased extraction of tocopherol from oil. Rim et al., [57] demonstrated that exposing peanut shells to infrared rays gives the highest antioxidant efficacy compared to conventional heating treatment. The antioxidant efficacy increases with infrared exposure time. In addition, Seok et al. [58] showed that when thermal processing of grapes using infrared was performed, the levels of antioxidants and phenolic compounds were increased. 

#### 3.2.5. Influence of Infrared Radiation on Microorganisms

Infrared radiation can be used to inhibit bacteria, spores, yeasts and mold in liquid and solid foods. The effectiveness of infrared inhibition depends on the amount of infrared energy, food temperature, wavelength, wave width, food depth, microorganism type, moisture content, and food material type. Increasing the capacity of the infrared source needed for heating produces more energy. Therefore, the total energy absorbed by microorganisms increases and thus increases microbial inhibition.

Hamanaka et al. [29] used infrared radiation to sterilize the grain surface of wheat and found that the surface temperature of wheat increased rapidly when infrared radiation fell on them without the need for conductors. When the radiation power was 0.5, 1, 1.5, and 2 kW, the temperatures within the device were 45, 65, 95, and 120 °C. As a result, the microbial content was 0.83, 1.14, 1.18, and 1.90 CFU/g after 60 s of exposure to infrared heating. Molin and Ostlund [59] studied the effect of infrared temperature on the inhibition of microorganisms. D values of *Basillus subtilis* were 26, 6.6, 9.3, and 3.2 s at 120, 140, 160, and 180 °C, respectively, while the z-value was 23 °C. The low treatment time at high temperatures was sufficient to eliminate pathogenic microorganisms. The logarithmic numbers of *E. coli* bacteria decreased to 0.76, 0.90 and 0.98 CFU/g after 2 min of infrared exposure [60].

Jun and Irudayaraj [61] used infrared within a wavelength of 5.88–6.66 µm using optical band bass filters to inhibit *Aspergillus niger* and *Fusarium proliferatum* in corn flour. The specific wavelength denatures the protein in microorganisms and results in a 40% increase in inhibition compared to the use of infrared radiation without determining a specific wavelength. If the wavelength was determined and not specified, the decrease in the logarithmic numbers of *A. niger* was 2.3 and 1.8 CFU/g, respectively, after five minutes of infrared radiation exposure. In contrast, the logarithmic numbers of *F. proliferatum* were 1.95 and 1.4 CFU/g, respectively, at infrared radiation exposure. The reason was that the energy absorption by the innate spores was greater at the elected wavelength and consequently lead to a higher mortality rate [61].

#### 3.2.6. Mechanism of Infrared and Microbial Inactivation 

Thermal inhibition works by damaging DNA, RNA, ribosome, cell cover, and proteins in bacterial cells. Sawai et al. [62] studied the mechanics of the microbiological inhibitor of infrared radiation against *E. coli* bacteria in saline phosphate fever. The obtained results suggested that partially damaged cells would become more sensitive to antibiotics that have an inhibitory action on the damaged part of the cell. RNA, proteins, and cell walls are more vulnerable to infrared heating than conductive heating. The order of magnitude of infrared damage is as follows:

Protein > RNA > Cell wall > DNA

Using infrared heating at 3.22 kW/m^2^ for 8 min resulted in a reduction of 1.8, 1.9, 2.7, and 3.2 log of *E. coli* when the Agar was rich in nalidixic, penicillin (PCG), rifampicin (RFG), and chloramphenicol (CP). However, the reduction rate of *E. coli* was 1.8 log without using any of above-mentioned antibiotics. This means that the effect of inhibitory factors led to a decrease of 0.1, 0.9 and 1.4 log due to PCG, RFP, and CP, respectively. The infrared penetration depth is a low. The surface temperature of the food materials increases rapidly and the heat is transferred to the food through thermal conduction. 

**Figure 8:**
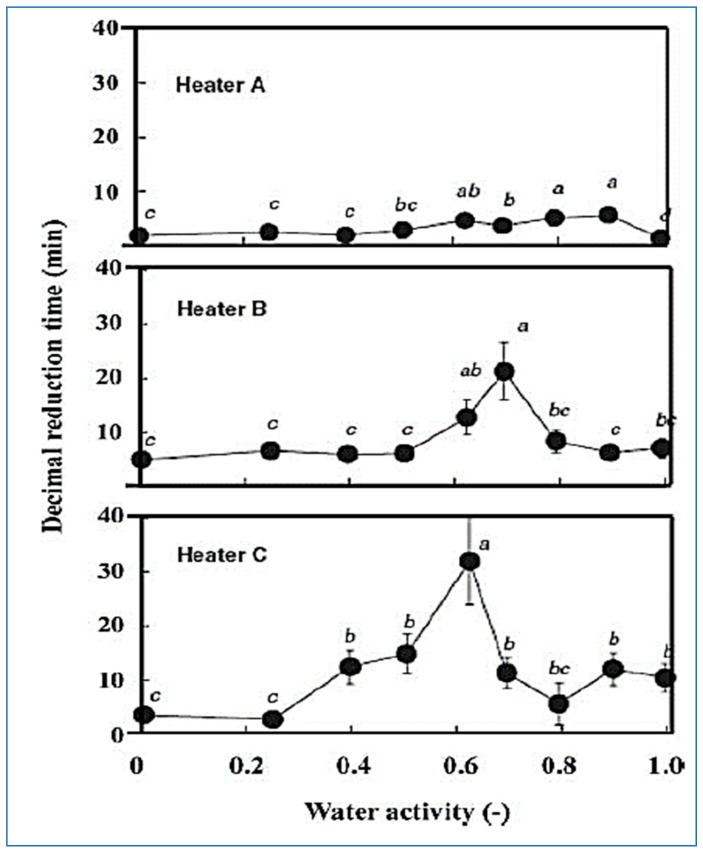
Relationship between water activity and decimal reduction times for *B. subtilis* spores using infrared treatment [28].

The thermal conductivity of solid foods is lower than liquid foods. In the case of liquid foods, heat transfer occurs by convection using infrared heating, thereby increasing the microbial mortality [2]. Hamanaka et al. [28] studied the inhibition efficiency of *B. subtilis* treated with three infrared heaters of different wavelengths (950, 1100, and 1150 nm). The results found that inhibition of pathogenic microorganisms at 950 nm was higher than other wavelengths at the same temperature. The decimal time at water activity of 0.7 and wavelengths of 950, 1100, and 1150 nm was 4, 12, and 22 min, respectively. The obtained results indicated that the inhibition efficiency was depended on the radiation spectrum, as shown in Figure 8. The effect of infrared radiation on microbial inhibition was decreased with increasing the food depth because infrared penetration depth is low, therefore, infrared can be used to sterilize food surfaces only. Rosenthal et al. [63] showed that infrared heating was efficient in reducing the growth of yeasts and molds on the surface of cheese at 70 °C for 5 min without affecting the quality of the cheese.

Infrared lamps used in hatching poultry eggs and pest control. According to Kirkpatrick [64], infrared rays led to the elimination of insects by 99% of *Sitophilus oryzae* and 93% of *Rhyzopertha dominica* and wheat temperature increased to 48.6 °C during treatment.

#### 3.2.7. Inhibition of Enzymes Using Infrared

Infrared radiation can be effectively used to inhibit enzymes. Lipooxygenase enzyme responsible for soybean damage is inhibited by 95.5% using infrared radiation [15]. Lipase and α amylases are strongly influenced by infrared radiation at a temperature of 30–40 °C [64,65]. Lipase activity decreases by 60% after infrared treatment for 6 min while it decreases by 70% after using thermal conductivity. The inhibition of the polyphenol oxidase enzyme in treated potato chips using infrared heating starts when the temperature of the center of the slice reaches 65 °C and the inhibition cannot reach 100% in the center of the slice. This requires the first area of the device to provide a higher capacity to ensure the inhibition of higher efficiency and reduce the thickness of the chips [62].

**Figure 9:**
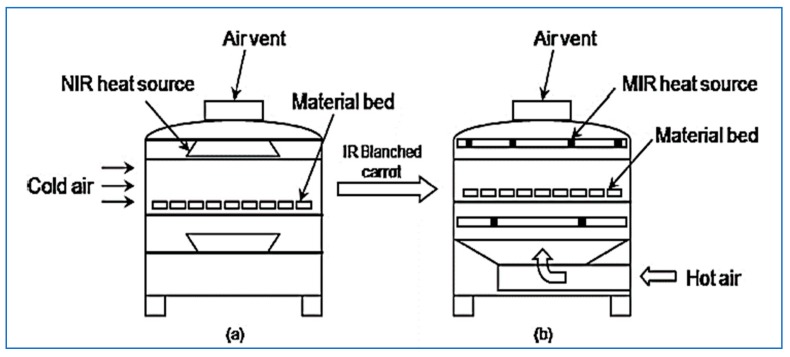
Schematic diagram of IR blanching (**a**) and hybrid drying (**b**) system [67].

Yi et al. [66] found that the best pre-treatment for apple cubes was dipping for 5 min in calcium chloride and ascorbic acid 0.5% for inhibition of brown coloration. Infrared heating at the intensity of 5000 W/m^2^ may inhibit the enzymatic polyphenol oxidase and peroxidase much faster than the intensity of 3000 W/m2. The enzymes polyphenol oxidase and peroxidase possessed high heat resistance and their inhibition process occurred by following the first-order kinetics and fractional-conversion models, respectively. Quick-boiling by using infrared drying is characterized by its rapid inhibition of complex enzymes that cause quality damage and no loss or very simple loss of vitamins, flavors, dyes, carbohydrates, and some water-soluble components. The reaction rate during infrared dry boiling is very low. The inhibition of phosphatase in infrared apple slices depends on the thickness of the chip and the intensity of the radiation. Infrared boiled peas retain more ascorbic acid and flavor than boiling with hot water. Infrared radiation can be used to inhibit enzymes effectively. The infrared boiling time of carrot slices requires a time of 10–15 min compared to the boiling steam and hot water methods, which requires a time of 5–10 min (Figure 9). This may be attributed to the gradual increase in the temperature of the product as a result of intermittent infrared heating and movement of air on the surface of the product. This led to the stability of the temperature of the product and improved the quality, where the amount of vitamin C was higher compared to the steam and hot water methods [67].

#### 3.2.8. Infrared Ovens and Baking

Baking bread is a complex process that involve a combination of physical, chemical and biochemical changes in foods such as gelatinization of starch, protein denaturation, release of carbon dioxide due to the addition of yeast, water evaporation, baking crust formation and brown reactions as a result of heat and mass transfer through the product and space inside the oven. Heat is transmitted to the dough by radiation, convection and conduction. Pei [68] classifies traditional bread into four phases: Crusty white bread, transfer heat from inside to crust, cooking or gelatinization and browning. The alternative technology for traditional bread is short-wave infrared [68,69,70].

In 1950, Ginzburg used infrared radiation as an oven to bake bread. At the time, this technique was not developed because of the lack of information about this technology. In 1970, researchers used infrared radiation as a means of heating food, especially for frying meat products [10,71]. Then, this technique was applied to baking bread [72]. Infrared biscuit bread was applied by Wade [70], and it was found that there is a wide range of biscuits that can be baked with an infrared wavelength of 1.2 µm and require half the time compared to the conventional method.

The benefit of using infrared heating in an oven for baking bread is to transfer the heat rapidly to the bread. The property of the bread allows a good penetration up to 2–3 mm and speed of heating. The reason why infrared ovens are better than conventional ovens is that this technique is more efficient in heating surfaces and central parts of food at a short baking time due to efficient heat transfer to the surface. This results in higher water content in the center of food during baking. Therefore, the shelf-life of the product will be better and longer [16]. 

**Figure 10:**
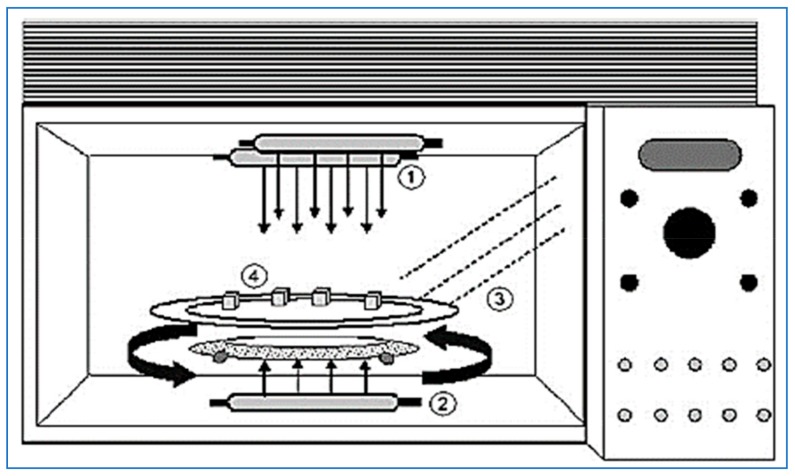
IR-microwave combination oven. (**1**) Upper halogen lamps, (**2**) lower halogen lamp, (**3**) microwaves, (**4**) turntable [76].

Heist and Cremer [73] studied the effect of infrared bread on the sensory qualities and energy use of cakes made from white and bleached and non-white flour and compared it to a traditional oven. Lee [74] merged between the microwave oven and the halogen lamp. Ninety percent of radiation energy within wavelength was less than 1 mµ and used as an infrared source. Two of them were used above and two at the bottom so that there was no interference between them in the microwave and this method gives more homogeneity in cooking. In this design, there were two mechanisms: Microwave heats food quickly, and infrared activates the reactions of tanning and crisping, and this method eliminates the problem of poor quality of baking using microwave [75]. The microwave has halogen lamps that emit infrared rays, which are divided into two parts, one part placed up and another down and there is a rotating base for the purpose of homogenization. The halogen lamps are 15 cm away from the material to be baked while the other halogen lamps are placed under the rotating plate (Figure 10). The results of the experiment are that the cake size increased with increasing baking time and the color and hardness of the cake were similar to the conventional oven [76].

#### 3.2.9. Infrared and Juices

**Figure 11:**
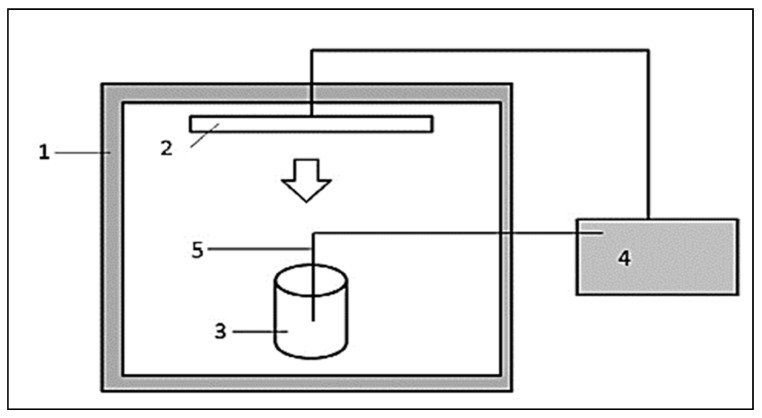
Schematic diagram of an infrared heater for the manufacture of lemon juice. (1) Heating chamber, (2) infrared emitter lamp, (3) juice filled bowl, (4) thermostat, (5) double thermostat [18].

**Figure 12:**
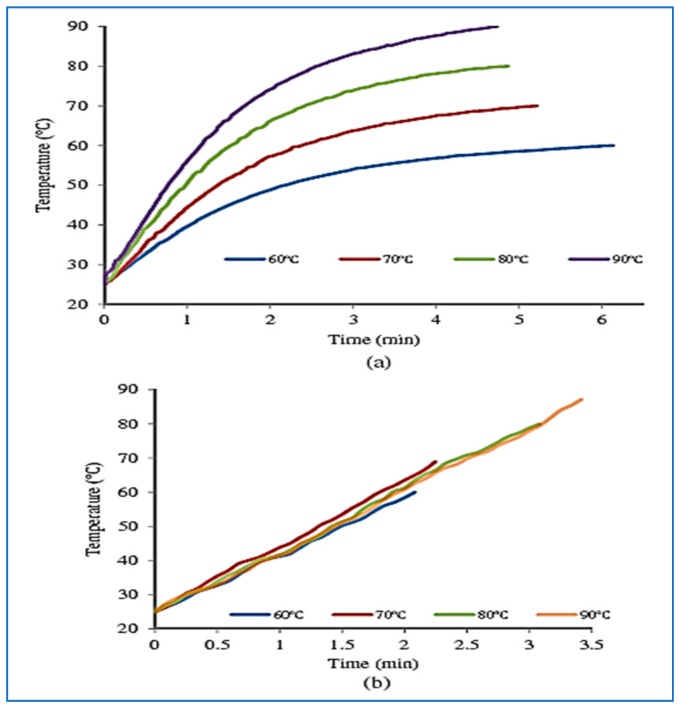
Juice temperatures change over time (**a**) conventional heating, (**b**) infrared heating [18].

Aghajanzadeh et al. [18] developed an infrared heating system for lime juice as shown in Figure 11. It consists of an infrared heating chamber of 1500 W. The distance between the infrared source and the surface of the juice is 8.5 cm and the system is equipped with a temperature control system. In addition, the system is equipped with a sample stirring system every 15 s for uniform heating. Figure 12 shows that the required time to reach temperature was lower using infrared radiation compared to conventional heating. This has positive effects on the nutritional quality of the juice and reduces the energy consumption and color of the juice. When the manufacturing temperature increases, the value of D (the time required to destroy 90% of ascorbic acid) decreases [32,77]. The temperature and heating time have a significant effect on the loss of ascorbic acid from the juice. Ascorbic acid is reduced by any heat treatment, whether infrared or conventional heating, and the process of crash of ascorbic acid follows the reaction kinetics during the process of juice production with a large correlation coefficient [18]. When the manufacturing temperature increases, the value of D (the time required to destroy 90% of ascorbic acid) decreases [32,77].

The retaining amount of ascorbic acid was higher using infrared heating compared to conventional heating, indicating that infrared heating is more effective in keeping juice during manufacturing [18].

#### 3.2.10. Infrared Drying of Fruits and Vegetables

In recent years, infrared drying technology has been successfully applied to fruits and vegetables, such as potato drying [78,79] sweet potatoes [80], onions [81,82], and apples [7,83]. Drying of seaweed, vegetables, fish flakes, and pasta was also examined using infrared tunnel dryers [84]. Bejar et al. [27] showed that the temperature of infrared drying had no significant effect on the surface, thickness, and size of the orange peel. It does not shrink when its moisture content drops to 0.1 kg water/kg d.b. However, a very simple contraction occurs when the temperature increases from 40 to 70 °C. The thickness of shrinkage was greater at 70 °C and lower at 40 °C. The volume of shrinkage was observed to be lower at 60 °C and higher at 50 °C due to the thickness of shrinkage. Shrinkage of infrared dried orange peels was the result of the amount of moisture evaporated.

Bejar et al. [27] also studied the effect of infrared drying temperatures on the color characteristics of orange peel (L *, a *, b *, C, and ΔE). There were significant differences in the color of dried orange peel compared to fresh samples. Infrared drying had a significant effect on a and b as the values of a, b and c decreased. Temperatures 50–60 had a significant effect on c and there was no significant effect of temperature 70 °C. The b value has decreased rapidly at 40, 50, and 60 °C and there was no significant effect at 70 °C. The L value was increased significantly using infrared drying. The color variation was the result of the breakdown of flavonoids and carotenoids, which were responsible for orange and yellow in crusts [85]. The lowest value of ΔE is obtained at the highest temperature. Infrared processing was applied to dry two varieties of strawberries. Two factors were used to find the optimized condition of infrared drying. The infrared time of *Camarosa* variety was 508, 280, and 246 min, while the infrared time of festival varieties was 536, 304, and 290 min at drying temperatures of 60, 70, and 80 °C, respectively. The results showed that infrared time was totally affected by the drying temperature. The drying time of *Cama-rosa* variety was longer than festival variety.

#### 3.2.11. Infrared Heating Cost

An et al. [86] reported the cost of using infrared heating compared to the diesel-burning air heater for the cultivation of strawberry. The average night air temperature was 6.6 °C in the infrared heater treatment and 7.1 °C in the air heater treatment. The results revealed that the heating cost of using the air heater system was $537.35 based on 543 L tax-free diesel, while the cost of using the infrared system was $203.05 by consuming 5685 kWh of electricity. Therefore, the infrared heater system was able to save approximately 62.2% of heating costs. The cost of different heating modes was calculated and summarized that the main cost of IR drying was the radiators. This study also presented a significant relationship between the costs of different types of emitters [87]. 

## 4. Conclusion and Future Aspects of IR

This review presented and showed that infrared technology was used in many food manufacturing processes, such as drying, boiling, heating, peeling, polyphenol recovery, freeze-drying, antioxidant recovery, microbiological inhibition, sterilization grains, bread, roasting of food, manufacture of juices, and cooking food. Infrared is an environmentally friendly thermal treatment and it is considered an advanced thermal process that can be exploited in food processing. This technique requires substantial in-depth studies and investigation into the possibility of using it in the manufacture of sensitive foods to the heat. Infrared in the future will be exploited to extract the essential oils, distillation of water and heating food by continuous methods, and expand their integration with other advanced techniques such as electric field, magnetic and ultrasonic thermal, in addition. Using IR is desirable to sterilize the food packaging with transparent packaging and studied the integration of radiation infrared with refractance window drying for the purpose of increasing energy efficiency. The effect of IR on physico-chemical properties, sensory properties, and nutritional values and as well as the interaction of food components under IR radiation may further justify the use of IR radiation as a future food processing option. The interaction between processes and products needs coherent experiments in order to gain more knowledge.

## Figures and Tables

**Table 1:** The infrared absorption bands characteristic of chemical groups relevant to the heating of food [3].

Relevant Food Component	Absorption Wavelength (μm)	Chemical Group
Water, sugars	2.7–3.3	Hydroxyl group (O–H)
Lipids, sugars, proteins	3.25–3.7	Aliphatic carbon-hydrogen bond
Lipids	5.71–5.76	Carbonyl group (C=O) (ester)
Proteins	5.92	Carbonyl group (C=O) (amide)
Proteins	2.83–3.33	Nitrogen-hydrogen group (–NH–)
Unsaturated lipids	4.44–4.76	Carbon-carbon double bond (C=O)

**Table 2:** Effect of FIR-radiation and heat treatments on total phenolic contents of water extract from peanut hulls [2].

Treatments	Time (min)						
	0	5	10	15	20	40	60
FIR-radiation	72.9^e^	79.3^de^	88.6^d^	99.4^cx^	107.8^cx^	124.1^bx^	141.6^ax^
Heat treatment	72.9^c^	79.8^b^	79.5^b^	78.6^by^	78.5^by^	86.7^ay^	90.3^ay^

^a–e^ different letters within a row are significantly different (*p* < 0.05); ^x–y^ different letters within a column are significantly different (*p* < 0.05).

**Table 3:** Total phenols and antioxidant efficacy of infrared dried jujube [53].

Parameters	Initial	Infrared (Standard) W			Infrared Capacity (Pre-Treated with 5% Potassium Carbonate and 0.5% Olive Oil for 2 min)		
		62	88	125	62	88	125
TPC (mg of GA/100 g of dry matter)	263.15^a^	181.6^e^	134.35^d^	221.24^b^	155.41^d^	191.32^c^	192.41^c^
DPPH (l mol trolox/100 g of dry matter)	4.23^a^	0.99^f^	1.98^c^	3.23^b^	1.51^d^	2.70^b^	2.55^c^

Values in the same line with different lowercase letters (a, b, c, d, e, f) are significantly different as per the Duncan test (*p* < 0.05).
